# The mechanisms of sorafenib resistance in hepatocellular carcinoma: theoretical basis and therapeutic aspects

**DOI:** 10.1038/s41392-020-0187-x

**Published:** 2020-06-10

**Authors:** Weiwei Tang, Ziyi Chen, Wenling Zhang, Ye Cheng, Betty Zhang, Fan Wu, Qian Wang, Shouju Wang, Dawei Rong, F. P. Reiter, E. N. De Toni, Xuehao Wang

**Affiliations:** 1 Department of General Surgery, Nanjing First Hospital, Nanjing Medical University, Nanjing, China; 2 Hepatobiliary/Liver Transplantation Center, The First Affiliated Hospital of Nanjing Medical University, Key Laboratory of Living Donor Transplantation, Chinese Academy of Medical Sciences, Nanjing, China; 3 Department of Gastroenterology, The First Affiliated Hospital of Nanjing Medical University, Nanjing, China; 4 Michael G. DeGroote School of Medicine, McMaster University, Hamilton, ON Canada; 5 Department of Radiology, The First Affiliated Hospital of Nanjing Medical University, Nanjing, China; 6 Department of Medicine II, University Hospital, LMU, Munich, Germany; 7 Liver Center Munich, University Hospital, LMU, Munich, Germany

**Keywords:** Drug development, Gastrointestinal cancer

## Abstract

Sorafenib is a multikinase inhibitor capable of facilitating apoptosis, mitigating angiogenesis and suppressing tumor cell proliferation. In late-stage hepatocellular carcinoma (HCC), sorafenib is currently an effective first-line therapy. Unfortunately, the development of drug resistance to sorafenib is becoming increasingly common. This study aims to identify factors contributing to resistance and ways to mitigate resistance. Recent studies have shown that epigenetics, transport processes, regulated cell death, and the tumor microenvironment are involved in the development of sorafenib resistance in HCC and subsequent HCC progression. This study summarizes discoveries achieved recently in terms of the principles of sorafenib resistance and outlines approaches suitable for improving therapeutic outcomes for HCC patients.

## Introduction

Hepatocellular carcinoma (HCC) is the second leading cause of cancer-related mortality globally and usually presents in patients with chronic liver inflammation associated with viral infection, alcohol overuse, or metabolic syndrome.^1,2^ Significant progress has been made in HCC prevention, diagnosis and treatment in the past. However, more than 50% of all HCC patients have a diagnosis at an advanced stage (Barcelona Clinic Liver Cancer stage B or higher), and 70% of patients relapse within the first 5 years of initial treatment.^2^ Early HCC is often resectable, but advanced HCC often requires sorafenib for systemic treatment in addition to local treatment with ablation, transarterial chemoembolization, or external irradiation.^3,4^

In a groundbreaking study, sorafenib, a multiple-target tyrosine kinase inhibitor (TKI) exhibited antiangiogenesis and antiproliferation effects and extended total median survival in advanced HCC patients.^5^ Sorafenib suppresses tumor cell proliferation by inhibiting Raf-1, B-Raf, and kinase activity in the Ras/Raf/MEK/ERK signaling pathways. In addition, sorafenib is capable of targeting platelet-derived growth factor receptor (PDGFR-β), vascular endothelial growth factor receptor (VEGFR) 2, hepatocyte factor receptor (c-KIT), and other proteins to inhibit tumor angiogenesis.^6^ In two significant clinical trials, Asia-Pacific and Sorafenib HCC assessment randomized protocol (SHARP), sorafenib was effective in improving the outcomes of HCC patients in the late stage, initiating a period of robust clinical research.^7,8^ Since 2017, one large phase III trial has suggested noninferiority of lenvatinib compared with sorafenib in the first-line setting. Furthermore, regorafenib, cabozantinib, and ramucirumab have received approval as second-line treatments after sorafenib.^9–12^ Checkpoint inhibitors have also opened new strategies for the treatment of HCC.^13,14^ Recently reported results from the IMbrave150 study (NCT03434379) show potential for the combination of atezolizumab with bevacizumab to expand the treatment options in first-line therapy for HCC.^15^ However, immunotherapy for HCC has not yet been approved in China or Germany. Sorafenib remains a cornerstone treatment in HCC that is supported by robust evidence and clinical experience.

Only approximately 30% of patients can benefit from sorafenib, and this population usually acquires drug resistance within 6 months.^16^ Adverse events identified in patients administered sorafenib mainly included gastrointestinal, physical or skin diseases (e.g., hand and foot skin reactions, weight loss, and diarrhea). In serious cases, sorafenib can cause high blood pressure and abdominal pain, leading to treatment discontinuation.^17^ Accordingly, the sorafenib resistance mechanisms should be clarified. Recent studies suggest a role of epigenetics, transport processes, regulated cell death, and the tumor microenvironment in the initiation and development of sorafenib resistance in HCC. This study summarizes discoveries achieved recently in terms of the principles of sorafenib resistance and outlines approaches suitable for improving therapeutic outcomes for HCC patients.

## Epigenetic regulation and sorafenib resistance in HCC

Epigenetic modifications can change the expression states of genes without changing DNA sequences, and some modifications can be inherited.^18^ In some cases, epigenetic changes are dynamic and respond to environmental stimuli. Epigenetic mechanisms regulate different physiological processes that occur in living organisms, including cell proliferation and differentiation.^19,20^ A deeper understanding of epigenetic modifications associated with HCC could provide the basis for developing innovative approaches to treat this disease. In this context, we will describe the different types of epigenetic mechanisms and their involvement in the resistance of HCC to sorafenib (Table 1).^21–53^

**Table 1:** Epigenetic regulation and sorafenib resistance in HCC

Molecules/drugs	Expression	Major effects	Pathway	Reference
*Non-coding RNAs*				
SNHG1(lncRNA)	Up	Contributing to SR by activating the Akt pathway and positively regulated by miR-21	Akt	^21^
NEAT1(lncRNA)	Up	Mediating SR by suppressing miR-335 expression, and dis-inhibition on c-Met-Akt signaling pathway	c-Met-Akt	^22^
H19 (lncRNA)	Down	Over-expression of H19 can reduce cell proliferation to reduce chemical resistance after sorafenib treatment	–	^23^
TUC338 (lncRNA)	Up	Functionally involved in SR hepatocarcinoma cells by targeting RASAL1	–	^24^
Ad5-AlncRNA	Down	Ad5-AlncRNA infected SR HCC cells will block miRNA function, inhibit PTEN down-regulation and AKT activation	PTEN/AKT	^25^
ROR(lncRNA)	Up	Sorafenib increases expression of ROR in vesicles inside and outside tumor cells, while siRNA to ROR increases sensitivity to chemotherapy	TGF-β	^26^
HOXA13(lncRNA)	Up	Stable over-expression of HOXA13 in liver cancer cell lines increases cancer cell proliferation and migration, and reduces its sensitivity to sorafenib	–	^27^
SNHG3(lncRNA)	Up	Inducing HCC cells EMT via miR-128/CD151 cascade activation	EMT	^28^
SNHG16(lncRNA)	Up	Functioning as an endogenous sponge for miR-140-5p and the effects of SNHG16 knockdown on SR could be blocked by miR-140-5p inhibitor	–	^29^
FOXD2-AS1(lncRNA)	Down	Over-expression of FOXD2-AS1 overcame the resistance of SR cells through functioned as a sponge for miR-150-5p to modulate TMEM9 expression	–	^30^
miR-27a	Up	Anti-miR-27a significantly increases protein expression of FOXO1 and PPAR-γ, increasing the efficacy of sorafenib	–	^31^
miR-374b	Down	Over-expression of miR-374b re-sensitizing HCC cells to sorafenib therapy by antagonizing PKM2-mediated glycolysis pathway	Glycolysis	^32^
miR-19a-3p	Up	Promoting tumor metastasis and chemoresistance through the PTENAKT pathway	PTEN/AKT	^33^
miR-199a-3p	Up	Inducing SR by activating rapamycin (mTOR) and p21 activated kinase 4 (PAK4),leading to the repression of FOXM1.	mTOR/PAK4	^34^
miR-494	Up	Over-expression increases cancer cell resistance to sorafenib via the mTOR pathway	mTOR	^35^
miR-137	Down	Upregulation of miR-137 reverses SR and cancer-initiating cell phenotypes by degrading ANT2	–	^36^
miR-221	Up	Modulating SR throughinhibition of Caspase-3-Mediated apoptosis	–	^37^
miR-125a-5p	Up	miR-125a inhibitors reduce the efficacy of sorafenib by interfering with the expression of matrix metalloproteinase 11, Zbtb7a proto-oncogene and c-Raf	–	^38^
miR-367-3p	Down	miR-367-3p may improve the efficacy of sorafenib by altering MDM2/AR/FKBP5/PHLPP/ (pAKT and pERK) signals	AKT/ERK	^39^
miR-181a	Up	Inducing SR through downregulation of RASSF1 expression	MAPK	^40^
miR-122	Down	Confering SR by targeting IGF-1R to regulate RAS/RAF/ERK signaling pathways	RAS/RAF/ERK	^41^
miR-591	Down	Over-expression of miR-591 inhibits FBP2 expression by blocking phosphoinositide 3-kinase/Akt/mammalian targets of the rapamycin axis, thereby inhibiting drug resistance	PI3K/AKT	^42^
miR-622	Down	Functionally targeting KRAS,whose inhibition markedly suppressed RAF/ERK and PI3K/AKT signaling and re-sensitized SR cells	RAF/ERK PI3K/AKT	^43^
miR-7	Down	Effectively silencing TYRO3 expression in SR cells, inhibiting TYRO3/growth arrest specific 6-mediated cancer cell migration and invasion	PI3K/AKT	^44^
*Methylation*				
BNIP3	–	Demethylation of BNIP3 promoter, but not histone acetylation, restored BNIP3 expression, driving resistant cells’ death	–	^45^
PRMT6	–	PRMT6 interferes with CRAF’s RAS / RAF binding potential, thereby altering ERK-mediated transport of PKM2 into the nucleus, reducing the tumorigenicity and sorafenib resistance of PRMT6 deficiency	PRMT6-ERK-PKM2 regulatory axis	^46^
PTK2	Up	PTK2 activates Wnt/β-catenin signaling by promoting the nuclear accumulation of β-catenin to activate CSC characteristics and drive the tumorigenicity of HCC cells, resulting in HCC recurrence and sorafenib resistance	Wnt/β-catenin signaling	^47^
5-AZA	–	5-AZA promotes the anticancer response by inhibiting the tumorigenicity of HCC cells and improves the response of sorafenib	–	^48^
MORC2	Up	The MORC2-NF2/KIBRA axis is critical for maintaining self-renewal, sorafenib resistance, and oncogenicity of HCC cells in vitro and in nude mice	Hippo	^49^
Shc3	Up	Demethylation-induced over-expression of Shc3 drives c-Raf-Independent activation of MEK/ERK in HCC	MEK/ERK	^50^
PD-L1	Up	Targeting the NFκB/PDL1/STAT3/DNMT1 axis can lead to dual inactivation of PD-L1 and DNMT1 inhibitors, reducing cancer cell resistance to sorafenib	NFκB/PDL1/STAT3/DNMT1 axis	^51^
–	–	Sorafenib causes methylation of oncogenes through BIRC3, FOXO3, MAPK3, SMAD2 and TSC2		^52^
MDIG	Up	MDIG affects the level of p21 (CIP1/WAF1) and the resistance of cancer cells to sorafenib through the expression of H3K9me3 in HCC	H3K9me3/p21	^53^
H19	Down	H19 expression was significantly downregulated in all six chemoresistant HCC cell lines. The promoter methylation of the H19 gene was significantly different in chemoresistant cell lines compared to their sensitive counterparts	–	^23^

### Noncoding RNA-based mechanisms

**Fig. 1:**
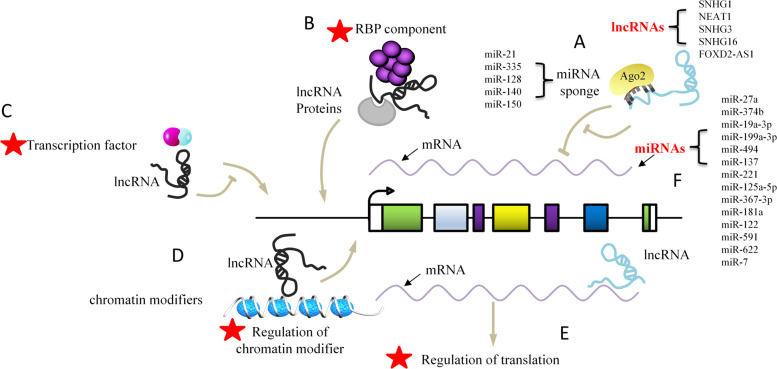
Molecular mechanisms by which lncRNAs and miRNAs modulate sorafenib resistance. **a** LncRNAs can act as a “sponge” of miRNAs, competitively binding miRNAs, and thus affect the regulation of miRNAs on downstream target genes. The figure lists the lncRNAs and corresponding sponged miRNAs associated with SR. **b** As a scaffold or bridge for protein interaction, lncRNAs affect the formation of protein multimers and regulate protein activity. **c** As an RNA decoy, lncRNAs bind to transcription factors and interfere with their binding to the gene promoter region, thereby regulating transcription. **d** LncRNAs recruit chromatin modifiers to alter the level of chromatin modification, thereby affecting gene transcription and expression. **e** LncRNAs bind to mRNA and inhibit translation. **f** MiRNAs have the ability to degrade mRNA and prevent mRNA translation. The figure lists the miRNAs associated with SR. Note: A pentagram indicates no relative report with SR in HCC

Increasing evidence indicates that noncoding RNAs (ncRNAs), including long noncoding RNAs (lncRNAs) and microRNAs (miRNAs), are critical for the development of sorafenib resistance in HCC (Fig. 1). Small nucleolar RNA host gene 3 (SNHG3) had significantly higher expression in highly metastatic HCC cells than in poorly metastatic HCC cells and induced epithelial–mesenchymal transition (EMT) through miR-128/CD151 cascade activation to produce sorafenib resistance.^28^ SNHG16 was reported to be upregulated in HepG2 sorafenib-resistant (SR) cells, and SNHG16 knockdown increased the sensitivity of HepG2 SR cells to sorafenib in vitro and in vivo. Mechanistic studies indicated that SNHG16 could be an endogenous sponge for miR-140-5p in HepG2 cells. Overexpression of miR-140-5p also made HepG2 SR cells more susceptible to sorafenib, and the influences exerted by SNHG16 knockdown on sorafenib resistance might be inhibited by miR-140-5p inhibitors.^29^ Overexpression of miR-591 has been reported to inhibit colony formation, as well as drug resistance, including sorafenib resistance, through the inhibition of the expression of far upstream elemental binding protein 2 (FBP2) via the phosphoinositide 3 kinase/Akt/mammalian target of the rapamycin axis.^42^ MiR-622 is significantly downregulated in HCC and functionally targets Kirsten rat sarcoma (KRAS), whose inhibition significantly inhibits RAF/ERK and PI3K/AKT signaling and resensitizes sorafenib-resistant cells.^43^ These studies indicate that ncRNAs may represent a medical treatment approach to overcome sorafenib resistance in HCC. However, further research is needed to determine whether ncRNAs are drivers or passengers in tumor progression, as numerous ncRNAs have been reported to be dysregulated in cancer progression. In addition, current studies have focused only on the role of lncRNAs as miRNA sponges in regulating target gene expression and mediating sorafenib resistance in HCC. Previous research indicates that lncRNAs have more mechanisms, such as binding to proteins regulating protein translation, interfering with the expression of genes encoding adjacent proteins, and forming complexes with proteins to regulate gene transcription, which are areas in need of further exploration.^54,55^ Further studies of clinical trials are urgently needed to promote ncRNA-based therapeutic interventions beneficial to HCC patients, which may offer treatment avenues for sorafenib resistance.

### Methylation

Methylation refers to the process of catalytically transferring methyl groups from active methyl compounds (such as S-adenosylmethionine) to other compounds. Examples consist of RNA methylation, histone methylation, and DNA methylation.^56^ Aberrant methylation causes gene expression to be changed, resulting in cancerous features.^57^ Wang et al.^49^ reported that the MORC2-NF2/KIBRA axis is critical to maintain sorafenib resistance and oncogenicity of HCC cells in vitro and in nude mice. MORC2 complexes with DNA methyltransferase 3A (DNMT3A) on the promoters of NF2 and KIBRA, causing DNA hypermethylation and transcriptional inhibition. Accordingly, under physiological and pathological conditions, NF2 and KIBRA are key targets of MORC2 for the regulation of fusion-induced Hippo signaling activation and contact inhibition of cell growth. Abeni et al.^52^ used medical chip technology to find 1230 differentially methylated genes in sorafenib-treated HA22T/VGH cells. After sorafenib treatment, oncogenes tend to be hypermethylated, while tumor suppressor genes tend to be hypomethylated. In addition, the lncRNA H19 is an example of maternal expression and epigenetic regulation of imprinted gene products and is believed to promote or inhibit tumors. In sorafenib-resistant cell lines, the promoter methylation of the H19 gene differs noticeably from that in sensitive cells. Overexpression of H19 sensitizes sorafenib-resistant cells by reducing cell proliferation after sorafenib treatment. A model of H19 knockout mice suggested that H19 promoted tumor progression and tumor cell proliferation after treatment with the carcinogen diethylnitrosamine (DEN), while administration of insulin-like growth factor 2 (IGF2) had no effect. Therefore, H19 may be a target for future strategies to overcome sorafenib resistance in HCC.^23^ Together, this evidence suggests that sorafenib may influence the methylation levels of cancer-related genes in HCC, which are valuable in tracing sorafenib resistance.

## Transport and sorafenib resistance in HCC

Sorafenib resistance involves ATP binding box (ABC) transporters, which reduce the effectiveness of chemotherapy by pulling drugs out of cancer cells and negatively affect the outcomes of anticancer therapies.^58,59^ In addition, exosomes are carriers of intercellular information and regulators of the tumor microenvironment. In normal cells, exosomes remove adverse biomolecules, but this mechanism may be hijacked in cancer cells. For example, drug-resistant cancer cells can encapsulate therapeutic drugs in exosomes and transport them out of tumor cells.^60,61^ The mechanisms of the transport process and sorafenib resistance in HCC are reviewed below (Table 2).^26,41,62–69^

**Table 2:** Transport and sorafenib resistance in HCC

Molecules/drugs	Expression		Major effects	Pathway	Reference
*ABC transporters*					
CRYO	–		CRYO’s ability to inhibit ABC pump and improve HCC cell response to sorafenib in non-toxic doses	–	^62^
CSN5	–		CSN5 silencing reverses sorafenib resistance of human hepatocellular carcinoma HepG2 cells	–	^63^
–	–		Changes in the expression of EMT regulatory proteins cause activation of the EMT process, so that HCC cells with sorafenib resistance have higher metastatic potential	EMT	^64^
*Exosomes*					
miR-744	Down	miR-744 can significantly inhibit the proliferation of HepG2 cells and reduce the resistance to sorafenib		–	^65^
Exosomes derived from HCC cells	–	exosomes derived from HCC cells induced SR by activating the HGF/c-Met/Akt signaling pathway and inhibiting sorafenib-induced apoptosis		HGF/c-Met/AKT	^66^
ROR(lncRNA)	Up	sorafenib increases the expression of ROR in tumor cells and extracellular vesicles, while siRNAs targeting ROR increase the efficacy of chemotherapy.		TGFβ	^26^
VLDLR(lncRNA)	Up	VLDLR gene knockdown reduced ABCG2 expression and increased sorafenib-induced cell death.		–	^67^
miR-122	–	AMSC transfected with miR-122 can effectively package miR-122 into secreted exosomes, making cancer cells sensitive to sorafenib		–	^41^
GRP78	Up	si-GRP78 modified exosomes combined with sorafenib was able to target GRP78 in HCC cells and inhibit the growth and invasion of the cancer cells		–	^68^
Dendritic cells pulsed with exosomes	–	Dendritic cells pulsed with exosomes in combination with PD-1 antibody increase the efficacy of sorafenib		–	^69^

### ABC transporters

Several TKIs, including sorafenib, were found to interact with ABC transporters.^58^ Such findings reveal a highly sophisticated situation in which TKIs are likely to act as substrates or inhibitors, depending on specific pump expression and the type of drug coadministered, its affinity for transporters, and its concentration. The repositioning of TKIs as ABC transporter antagonists opens up new avenues for anticancer therapy and clinical strategies aimed at counteracting drug resistance. Di Giacomo et al.^62^ investigated the ability of CRYO (the natural sesquiterpene component of many essential oils) to inhibit ABC pumps and improve the response of HCC cells to sorafenib at nontoxic doses. They obtained a clonal subfamily from human HCC cells exhibiting increased multidrug resistance (MDR) related to upregulation of MRP1 and MRP2. In addition, CRYO restrained sorafenib degradation, facilitated its intracellular accumulation and strengthened its cytotoxic response. Another study reported that COP9 signaling corset 5 (CSN5) was associated with sorafenib resistance in HepG2/S HCC cells. After CSN5 silencing, resistance to sorafenib was reversed, and several resistance-related proteins (including ABCB1, ABCC2, and ABCG2) were downregulated.^63^ Furthermore, ABCC1-3 expression increased in SR cells. Enhanced SR cell migration and invasion and an increased ratio of CD44+ to CD44+ CD133+ cells were observed in SR cells.^64^

### Exosomes

**Fig. 2:**
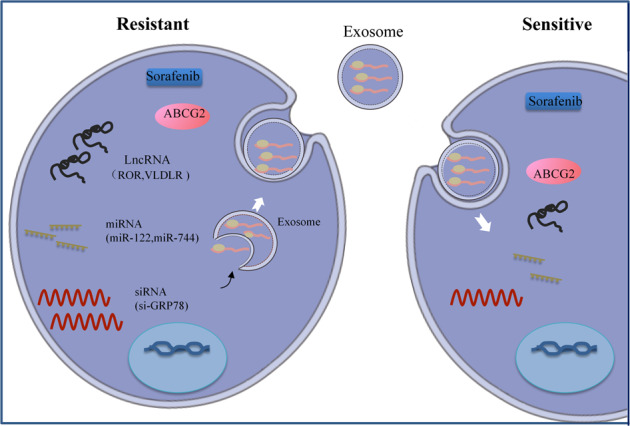
Exosome application in HCC therapy. Exosomes derived from cancer cells can be used to deliver functional RNAs, including lncRNAs, siRNAs, miRNAs, and mRNAs

Exosomes, which are small extracellular vesicles (EVs), contribute to cell-to-cell communication and have emerged as a therapeutic target.^70–72^ LincRNA-VLDLR (linc-VLDLR) showed significant upregulation in malignant liver cells. Exposure of HCC cells to various anticancer agents (e.g., sorafenib) increased the expression of linc-VLDLR in cells and in EVs released from these cells. Incubation with EVs downregulated chemotherapy-triggered cell death and increased linc-VLDLR expression in recipient cells. In addition, knockdown of linc-VLDLR reduced ABCG2 (ATP binding cassette, subfamily G member 2) expression, and overexpression of this protein reduced the effect of linc-VLDLR knockdown on sorafenib-induced cell death.^67^ Apart from lncRNAs, miRNAs can also be carried in exosomes, and miR-122-transfected adipose tissue mesenchymal stem cells (AMSCs) have been shown to efficiently package miR-122 in secreted exosomes, probably mediating AMSC and HCC cell miR-122-related communication processes, thus making HCC cells sensitive to sorafenib by altering the expression of miR-122 target genes. Intratumor injection of miR-122-exo noticeably improved the antitumor performance of sorafenib in HCC in vivo.^41^ Li et al.^68^ showed that the combination of si-GRP78-modified exosomes and sorafenib could target GRP78 in HCC cells and restrain cancer cells from growing and invading. Therefore, si-GRP78-altered exosomes can sensitize sorafenib-resistant cancer cells to reverse drug resistance. Dendritic cells (DCs) are critical to both primary and secondary immune responses; thus, DC-derived exosomes are candidates for specific cancer treatment. Shi et al.^69^ found that regulatory T cells in tumor tissues and sorafenib treatment of HCC mice in situ increased programmed death ligand 1 (PD-L1) expression. When DCs were pulsed with exosomes from tumor cells, the number of regulatory T cells decreased, and the number of CD8^+^ T cells increased. When anti-programmed death receptor-1 (PD-1) antibody (PD-1 Ab) was added, the exhausted CD8^+^ T cells were restored, while the regulatory T cell number remained unchanged. Because exosomes are involved in the development of many diseases, researchers have used exosomes as a therapeutic strategy, in which exosomes are loaded with therapeutic agents such as functional proteins, ncRNAs, and chemotherapy drugs (Fig. 2). The resistance mechanisms involving exosome-mediated crosstalk need to be addressed by future therapeutic strategies for advanced-stage HCC.

## Regulated cell death (RCD) and sorafenib resistance in HCC

RCD is used to describe the death of cells originating from the intracellular or extracellular microenvironment via molecular mechanisms when other adaptive responses cannot restore cell homeostasis, and RCD can be divided into different categories, including apoptosis, autophagic cell death, proptosis, ferroptosis, etc., according to the different mechanisms.^73,74^ It has been reported that RCD, especially autophagy and ferroptosis, is involved in sorafenib resistance in HCC (Table 3).^75–91^

**Table 3:** Regulated cell death and sorafenib resistance in HCC

Autophagy				
IRE1		Sorafenib induces apoptosis and autophagy through endoplasmic reticulum stress, and further induces autophagy independently of the MEK1/2-ERK1/2 pathway.	MEK1/2-ERK1/2	^75^
SHP-1		Silencing of SHP-1 by small interference RNA (siRNA) reduced the effect of sorafenib on P-STAT3 and autophagy	STAT3/Mcl-1/Beclin 1	^76^
AKT		Inhibition of Akt reversed the acquired resistance to sorafenib by switching autophagy from a cytoprotective role to a death-promoting mechanism	–	^77^
HDACIs	–	Histone deacetylase inhibitors HDACIs sensitized HCC cells to sorafenib treatment by regulating the acetylation level of Beclin-1	P53	^78^
Melatonin	–	Melatonin increased the sensitivity of HCC to sorafenib by inhibiting autophagy through the PERK-ATF4-Beclin1 pathway	PERK/ATF4/Beclin1	^79^
PSMD10	Up	PSMD10/gankyrin induced autophagy to induce SR through cytoplasmic interaction with ATG7 and nuclear transactivation of ATG7 expression	–	^80^
	–	Sorafenib substantially increased phosphorylation of AMPK and consequently autophagy in Huh7	AMPK	^81^
ADRB2	Up	ADRB2 signals negatively regulate autophagy, stabilize HIF-1α, reprogram glucose metabolism in HCC cells, and acquire resistance to sorafenib	ADRB2	^82^
BCLAF1	Up	High expression of BCLAF1 might contribute to SR in HCC patients	–	^83^
SNHG1(lncRNA)	up	Contributing to sorafenib resistance by activating the AKT pathway and its nuclear expression is promoted by miR-21	AKT	^21^
Capsaicin	–	Capsaicin and sorafenib combination treatment inhibited the growth, invasion and metastasis of HCC cells and induced autophagy in a synergistic manner	EGFR PI3K/Akt/mTOR	^84^
CD24	Up	CD24-related sorafenib resistance was accompanied by the activation of autophagy and can be blocked by the inhibition of autophagy	mTOR/AKT	^85^
miR-21	Up	Inhibiting miR-21 enhances the efficacy of sorafenib in the treatment of sorafenib-resistant HCC tumors, and reduces sorafenib resistance	AKT/PTEN	^86^
miR-423-5p	Up	Promoting autophagy in cancer cells and was increased in serum from HCC patients treated with sorafenib	–	^87^
*Ferroptosis*				
AIFM2	–	AIFM2 blocks ferroptosis independent of ubiquinol metabolism.	–	^88^
ZFP36/TTP	–	RNA-binding protein ZFP36/TTP protects against ferroptosis by regulating autophagy signaling pathway in hepatic stellate cells	–	^89^
MT-1G	–	MT-1G enhances the anticancer activity of sorafenib. MT-1G inhibition by RNA interference increases glutathione depletion and lipid peroxidation, which contributes to sorafenib-induced hypertrophy	–	^90^
ELAVL1	–	ELAVL1 promotes autophagy activation by binding to AU-rich elements in the 3′ untranslated region F3 of BECN1/Beclin1 mRNA. Sorafenib treatment can reduce liver fibrosis in rats by inducing hepatic stellate cell (HSC) hypertrophy	–	^91^

### Autophagy

**Fig. 3:**
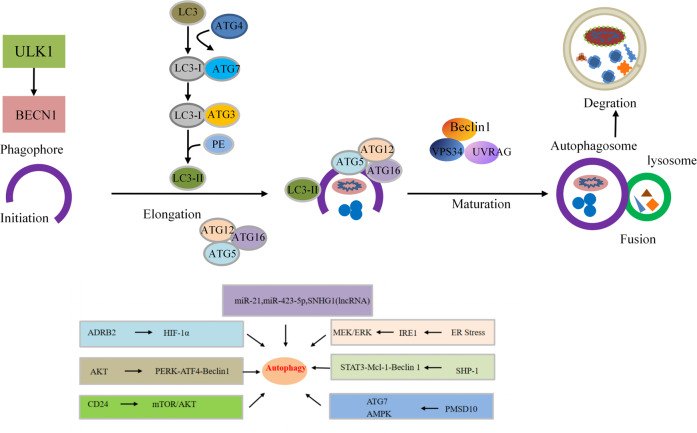
Schematic diagram of autophagy flux. Autophagy is initiated (primed) by the nucleation of a membrane or phage. This process is initiated by the ULK1-Atg13-FIP200 complex. The membrane is then elongated to engulf the cytoplasmic component (elongation). The elongation of the phagocytic membrane depends on the Atg5-Atg12-Atg16 and LC3 coupling systems. At a later stage of autophagosome formation, LC3-II is localized to the elongated barrier membrane, while the Atg5-Atg12-Atg16 complex dissociates therefrom. Finally, the barrier membrane is closed to form autophagosomes. After autophagosome formation, lysosomes fuse with autophagosomes to form autolysosomes (autophagosome–lysosome fusions). The lysosomal hydrolase degrades the content (degradation) in the autophagosome. Beclin 1-VPS34-UVRAG complex positively regulates fusion and degradation

Autophagy is an important process leading to intracellular material turnover in eukaryotes. Some of the damaged proteins or organelles in this process are encapsulated in bilayered autophagic vesicles and sent to lysosomes for degradation and recycling.^92,93^ The effect exerted by autophagy in cancer cells plays a double-edged sword.^94,95^ Basic autophagy is a cancer suppressor that maintains genomic stability in normal cells, whereas activated autophagy promotes the survival of cancer cells under stress once cancer occurs.^96^ Autophagy is also thought to be an important mechanism for drug resistance by supporting the survival of tumor cells in the case of therapeutic and metabolic stress.^97^ Therefore, it is necessary to determine how to control cell growth, apoptosis and sorafenib resistance by changing autophagy levels, which could possibly improve the efficacy of sorafenib and the treatment of HCC. Activation of Akt is considered to account for the mediation of acquired resistance to sorafenib. Zhai et al.^77^ found that Akt inhibition reversed acquired resistance to sorafenib by transforming autophagy from a cell-protective effect to a system that promotes cell death. Moreover, sorafenib effectively reversed the activation of metformin-induced mTORC2 and enhanced the inhibitory effect of metformin on the mTORC1 and MAPK pathways in HCC cells. Pharmacological inhibition of autophagy sensitized HCC cells to apoptosis induced by metformin and sorafenib.^79^ Studies have also shown that patients exhibiting high ATG7 expression and an active autophagy state have poor prognosis after sorafenib treatment.^80^ Due to different autophagy responses to sorafenib, different HCC cell lines have different sensitivities to sorafenib.^81^ Animal models are seen as key tools in cancer research. A recent study has shown that ADRB2 signaling restrained autophagy through disruption of the Beclin1/VPS34/Atg14 complex in an AKT-dependent manner, causing HIF1α stabilization, reprogramming of glucose metabolism in HCC cells, and sorafenib resistance in DEN-induced HCC mouse models.^82^ Despite ample evidence that targeted autophagy processes represent potential therapeutic interventions for HCC (Fig. 3), there are many unresolved issues related to autophagy in sorafenib resistance. How do we selectively target autophagy mechanisms? How do we balance the dual effects of autophagy? What determines the threshold for autophagy to change from a survival mechanism to one that promotes apoptosis or autophagic death? The answers to these questions need to be further elucidated through research.

### Ferroptosis

**Fig. 4:**
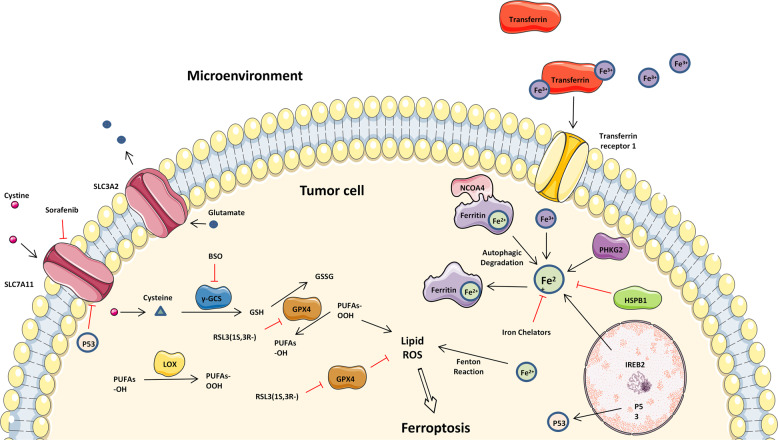
The mechanisms of ferroptosis and sorafenib resistance. The key factor leading to ferroptosis are ROS, which are produced by iron accumulation and lipid peroxidation. This figure shows the relevant pathways that regulate iron and lipid peroxidation. SLC3A2 solute carrier family 3 member 2, SLC7A11 solute carrier family 7 member 11, BSO L-buthionine-sulfoximine, GPX4 glutathione peroxidase 4, PUFA polyunsaturated fatty acids, LOX lipoxygenase, NCOA4 nuclear receptor coactivator 4, PHKG2 phosphorylase kinase G2, HSPB1 heat shock protein family B (small) member 1, IREB2 iron-responsive element-binding protein 2

Ferroptosis refers to a nonapoptotic, RCD procedure involving the abnormal metabolism of lipid oxides in cells catalyzed by iron ions or iron enzymes and has been identified recently. In this process, various inducers break the cell redox balance and produce considerable lipid peroxidation products, thereby triggering cell death. A growing body of research shows that the relationships between ferroptosis and cancer are significantly complex and that ferroptosis holds promise as a novel cancer treatment.^98^ The fact that sorafenib induces ferroptosis, which promotes sorafenib resistance adds to the complexity of sorafenib’s antitumor mechanism in HCC (Fig. 4).^98^ A recent study reported that the depletion of intracellular iron stores realized through the iron chelator deferoxamine (DFX) strikingly protected HCC cells from the cytotoxic effects of sorafenib. Moreover, they identified that DFX did not prevent sorafenib from reaching its intracellular target kinases. Instead, the depletion of intracellular iron stores prevented sorafenib from inducing oxidative stress in HCC cells.^99^ Beyond that, Lachaier et al.^100^ reported that sorafenib induced ferroptosis in different cancer cell lines. Compared to other kinase inhibitors, sorafenib was the only drug that displayed ferroptotic efficacy. From a mechanism of action perspective, Sun et al.^90^ found that metallothionein (MT)-1G was a critical regulator and promising therapeutic target of sorafenib resistance in human HCC. The expression of MT-1G messenger RNA and protein was noticeably triggered by sorafenib but not by other clinically relevant kinase inhibitors. The activation of the transcription factor nuclear factor erythroid 2-related factor 2, but not of p53 and hypoxia-inducible factor 1-alpha, was critical to induce MT-1G expression following sorafenib treatment. The molecular mechanisms of MT-1G in sorafenib resistance participate in the suppression of ferroptosis, a novel form of altered cell death. Knockdown of MT-1G by RNA interference increased glutathione depletion and lipid peroxidation, which contributed to sorafenib-induced ferroptosis. According to another study, ELAVL1 upregulation, ferritinophagy activation, and ferroptosis induction occurred in primary human hepatic stellate cells (HSCs) from the collected human liver tissues.^91^ In conclusion, sorafenib-induced ferroptosis may be an effective mechanism for inducing HCC cell death, regardless of its inhibitory effect on kinases.

## Tumor environment and sorafenib resistance in HCC

Cancer metastasis, invasion and growth are affected by the tumor microenvironment, which is comprised of various nonmalignant stromal cells. A sophisticated and multidirectional interplay between immune or nonimmune stromal cells and tumor cells during HCC development and progression has been recently shown (Table 4).^101–122^

**Table 4:** Tumor environment and sorafenib resistance in HCC

Hypoxia				
EF24	–	Hypoxia induced by sustained sorafenib treatment confers sorafenib resistance to HCC through HIF-1α and NF-κB activation	HIF-1α NF-κB	^101^
Melatonin	–	Melatonin enhances sorafenib actions in hepatocarcinoma cells by inhibiting mTORC1/p70S6K/HIF-1α and hypoxia-mediated mitophagy	mTORC1/p70S6K/HIF-1α	^102^
miR-338-3p	Down	Inhibiting hepatocarcinoma cells and sensitized these cells to sorafenib by targeting hypoxia-induced factor 1α	HIF-1α	^103^
Genistein	–	Genistein suppresses aerobic glycolysis and induces HCC cell death	GLUT1, HK2	^104^
HIF -2α	Up	Targeting hypoxia-inducible factor-2α enhances sorafenib antitumor activity via β-catenin/C-Myc-dependent pathways	β-catenin/C-Myc	^105^
2ME2	–	2ME2 reduces the expression of HIF-1α and HIF-2α and its downstream molecules, increases the sensitivity of hypoxia HCC cells to it, and inhibits the nuclear transport of HIF-1α and HIF-2α proteins	HIF-1α HIF-2α	^106^
HIF-2α	Up	Upregulation of HIF-2α induced by sorafenib contributed to the resistance by activating the TGF-α/EGFR pathway	TGF-α/EGFR	^107^
PT-2385	Up	HIF-2α inhibitor, PT-2385 significantly enhanced sorafenib efficacy by suppressing HIF-2α, increasing AR and suppressing downstream pSTAT3/pAKT/pERK pathways	pSTAT3/pAKT/pERK	^108^
Metformin	–	Metformin and insulin impact on clinical outcome in patients with advanced hepatocellular carcinoma receiving sorafenib	–	^109^
PRMT6	Up	PRMT6 drives glycolysis through the transcriptional repressor REST as a new target of hypoxia, and 2DG inhibits glycolysis, reversing PRMT6 deficiency-mediated tumorigenicity and sorafenib resistance in HCC	PRMT6-ERK-PKM2	^46^
RIT1	Up	RIT1 deficiency increased drug sensitivity to sorafenib treatment.HIF-1α directly transcriptionally upregulated RIT1	HIF-1α	^110^
PFKFB3	Up	HIF-1α deficiency impaired sorafenib resistance induced by PFKFB3	HIF-1α	^111^
PP2A	Up	LB-100 is a PP2A inhibitor that sensitizes HCC cells to sorafenib in a hypoxic environment. PP2A inactivation mediates this effect, leading to increased levels of p-Smad3	Smad3	^112^
*Immune microenvironment*				
CCR2	Up	Natural CCR2 antagonists can enhance the efficacy of low-dose sorafenib by increasing the number of CD8 ^+^ T cells in tumors and increasing the death of tumor cells	–	^113^
CXCR4	Up	CXCR4 inhibition in tumor microenvironment facilitates anti-programmed death receptor-1 immunotherapy in sorafenib-treated hepatocellular carcinoma in mice	PD-1	^114^
HGF	Up	HGF chemoattracts more macrophages migrated from surrounding area, regulates the distribution of M2 macrophages and increases hepatoma resistance to sorafenib in a feed-forward manner	HGF/c-Met, ERK1/2/MAPK, PI3K/AKT	^115^
CCL2, CCL17	Up	Tumor-associated neutrophils recruit macrophages and T-regulatory cells to promote progression of HCC and resistance to sorafenib	–	^116^
PD-1	Up	Anti-PD-1 immunotherapy might complement sorafenib in treating HCC patients by targeting sorafenib-resistant cancer cells, and the dual pERK and PD-1 biomarkers would help HCC patient selection to achieve optimal clinical benefits	ERK	^117^
–	–	sorafenib attenuated the function of natural killer cells infiltrated in HCC through inhibiting ERK1/2	ERK1/2	^118^
YB-1	Up	YB-1 expression was upregulated in chemoresistant HCC cells, and YB-1 knockdown reversed SR via T-cell activation in the tumor microenvironment due to blocked PD-L1 expression	–	^119^
*Virus reactivation*				
MYH9		Targeting MYH9 can improve the sensitivity of sorafenib to liver cancer cells in vivo through the MYH9/GSK3β/β-catenin/c-Jun feedback loop	MYH9/GSK3β/β-catenin/c-Jun	^120^
pMAPK14		Decreasing pMAPK14 can improve the therapeutic efficacy of sorafenib through the Raf-Mek-Erk pathway	Raf-Mek-Erk	^121^
HBx-ΔC1		HBx-ΔC1 enhances liver CSCs self-renewal, tumorigenicity, chemoresistance, and resistance to sorafenib through Stat3/Nanog cascade	Stat3/Nanog	^122^

### Hypoxia

**Fig. 5:**
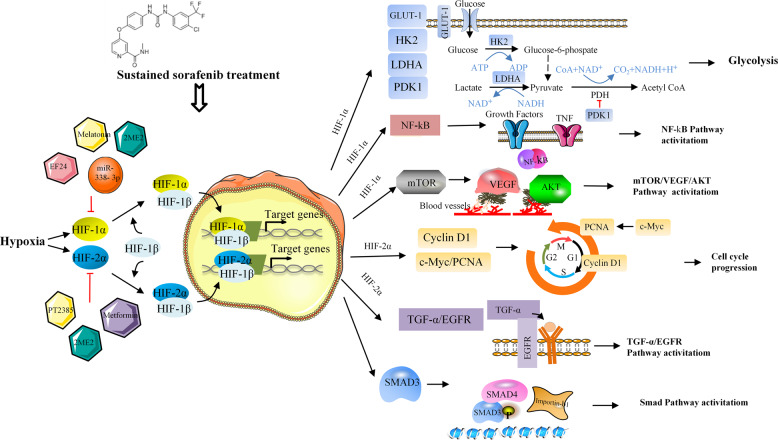
Hypoxia-related sorafenib resistance mechanisms and strategies for targeting HIFs. Sustained sorafenib treatment enhances hypoxia-inducible factors 1 alpha and 2 alpha, thereby promoting transcription of multiple genes involved in proliferation, glucose metabolism, angiogenesis, and different pathways, leading to sorafenib resistance. This resistance can be overcome by different small molecules or drugs inhibiting HIFs

Hypoxia in HCC drives angiogenesis through a vascular endothelial growth factor (VEGF)-producing process and hypoxia-inducible factor 1 (HIF-1α) activation. Therefore, the antiangiogenic effect of sorafenib results from blockade of the HIF-1α/VEGF pathway.^123,124^ Sorafenib suppresses the synthesis of hypoxia-inducible factor 1 (HIF-1α), resulting in a decrease in VEGF expression and tumor angiogenesis in HCC.^125^ In addition, acquired sorafenib resistance and an anoxic microenvironment show an interesting correlation. Continuous sorafenib treatment results in inhibition of the tumor’s antiangiogenic activity and subsequent hypoxia within the tumor, which facilitates the selection of resistant cell clones to adapt to oxygen and nutrition deficits, thereby limiting the efficiency of sorafenib. A study by Liang et al.^101^ reported that hypoxia induced by continued sorafenib treatment conferred sorafenib resistance in HCC via HIF-1α and NF-κB activation. EF24, a molecule having structural similarity to curcumin, overturned sorafenib resistance via VHL-dependent HIF-1α degradation and NF-κB inactivation. Prieto-Domínguez et al.^106^ found that the coadministration of melatonin and sorafenib decreased the expression of HIF-1α mitophagy targets and restrained the formation of autophagosomes and subsequent colocalization of mitochondria and lysosomes. Melatonin enhanced sensitivity to sorafenib in Hep3B cells and blocked the synthesis of HIF-1α, thus preventing the protective cell phagocytosis caused by the hypoxic microenvironment, which is an important part of the multifactor mechanism responsible for the failure of chemotherapy. In addition, miR-338-3p was shown to inhibit HCC tumor growth and sensitize HCC cells to sorafenib by downregulating HIF-1α.^103^ Galectin-1 has been reported as a predictive marker of sorafenib resistance and a downstream target of the AKT/mTOR/HIF-1α signaling pathway.^126^ Considering the feedback system between HIF-1α and HIF-2α subunits, we believe that sorafenib treatment is likely to upregulate HIF-2α by inhibiting HIF-1α, enhancing sorafenib resistance and tumor growth.^105,127^ It has also been confirmed that the increase in HIF-2α triggered by sorafenib contributes to resistance through the activation of the TGF-α/EGFR pathway.^107^ Another study found that the HIF-2α inhibitor PT-2385 significantly enhanced sorafenib efficacy by suppressing HIF-2α, increasing androgen receptor (AR) and suppressing downstream pSTAT3/pAKT/pERK pathways.^108^ These studies endorse the existing relationship between high HIF expression and resistance to sorafenib (Fig. 5), demonstrating that hypoxia evidently impacts sorafenib therapy and suggesting that inducing hypoxia is a promising approach to overcoming resistance.

### Immune microenvironment

**Fig. 6:**
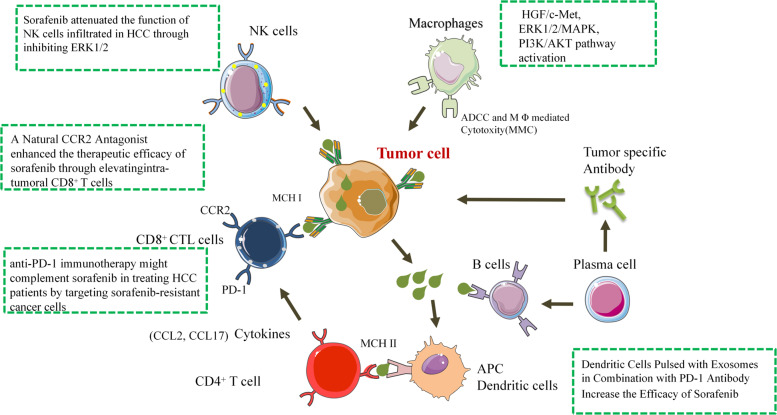
Schematic diagram of the sorafenib resistance-mediated immune mechanism. CD8^+^ CTL cells, NK cells, DC cells, and macrophages have been confirmed to be involved in sorafenib resistance through different mechanisms

Tumor immune escape has garnered interest in the development of tumor immunotherapy strategies, which have proved to be effective for some malignant neoplasms.^128^ Tumor-associated macrophages (TAMs) restrain antitumor immunity and facilitate tumor progression by expressing cytokines and chemokines.^128^ Current clinical trials of therapeutic drugs that promote phagocytosis or inhibit survival, proliferation, transport or polarization of TAMs have shown improved tumor prognosis. In HCC, Yao et al.^113^ reported that a natural product from Abies georgei, which is termed 747 and is related in structure to kaempferol, exhibited sensitivity and selectivity as a CCR2 antagonist. 747 enhanced the therapeutic efficacy of low-dose sorafenib without obvious toxicity by elevating the numbers of intratumoral CD8^+^ T cells and increasing the death of tumor cells. Chen et al.^114^ found that sorafenib increased the numbers of F4/80^+^ TAMs and CD11b^+^Gr-1^+^ and CD45^+^CXCR4^+^ myeloid cells in both HCA-1 and JHH-7 HCC models. Moreover, sorafenib treatment resulted in an increase in the fraction of tumor-infiltrating CD4^+^CD25^+^FoxP3^+^ regulatory T cells (Tregs) in HCA-1 tumors. Using AMD3100 to inhibit the stromal cell-derived 1 receptor (C–X–C receptor type 4 or CXCR4) can prevent polarization of the immunosuppressive microenvironment after sorafenib treatment, inhibit tumor growth, reduce lung metastasis, and improve survival. In addition, studies have demonstrated that M2, but not M1, macrophages maintain tumor growth and metastasis by secreting hepatocyte growth factor (HGF), thereby significantly increasing tumor resistance to sorafenib. HGF activates the HGF/c-Met, ERK1/2/MAPK and PI3K/AKT pathways in tumor cells. In vivo M2 tumor-associated macrophages accumulate more in sorafenib-resistant tumors than in sorafenib-sensitive tumors and produce large amounts of HGF. HGF can attract more macrophages from the surrounding area and regulate M2 macrophage distribution and feedback to enhance the sorafenib resistance of liver cancer.^115^ Neutrophils are capable of promoting or inhibiting tumor progression via the release of cytokines, which is determined by the tumor microenvironment. Factors generated by tumor-associated neutrophils (TANs) and their effects on tumor progression have not been elucidated. Zhou and colleagues^116^ explored the roles of TANs in the progression of HCC using cell lines and immune cells isolated from patients. They found that in tumor-bearing mice, sorafenib increased the number of TANs as well as CCL2 and CCL17 levels in the tumor. HCC tissues treated with sorafenib before surgery contained more TANs than tissues not treated with sorafenib. In mice, TAN depletion and sorafenib administration suppressed tumor growth and neovascularization more significantly than sorafenib administration alone. Phosphorylated extracellular signaling-regulated kinase (pERK) has been proposed as a marker for predicting the response to sorafenib in HCC, but clinical support is mixed or even contradictory. Chen et al.^117^ found that the pERK expression level varied in different patients with liver nodules. Mouse and human HCC samples with low pERK expression showed noticeable increases in intratumor CD8^+^ cytotoxic T lymphocytes with robust inflammatory infiltrating cells and expression of PD-1, suggesting that anti-PD-1 immunotherapy may supplement sorafenib in HCC patients by targeting sorafenib-resistant cancer cells and overcoming drug resistance. Zhu et al.^129^ reported a patient with advanced HCC who had a number of lung metastases that progressed during sorafenib treatment. SHR-1210 (an anti-PD-1 therapy) alone was used as a second-line treatment. Although lung metastasis did not decrease after 3 months of treatment, it declined noticeably and partially disappeared after 6 months of treatment. In addition, all lung metastases decreased continuously even after 17 months of treatment. Alpha-fetoprotein levels revealed a similar effect. After 19 months of follow-up, the patient was in good health. This suggests that SHR-1210 alone as a second-line therapy for HCC patients has a good antitumor effect. Modifying the tumor microenvironment with immune checkpoint blockade is an emerging and promising strategy in the field of HCC treatment (Fig. 6). The concept of immune checkpoint blockade is now being investigated to treat advanced tumors in adjuvant and neoadjuvant settings.

### Viral reactivation

Chronic hepatitis B virus (HBV) and hepatitis C virus (HCV) infection are major causes of HCC in the Western and Eastern regions.^130^ The reactivation process of virus infection in the setting of chemotherapy and immunosuppression is likely to cause fulminant liver failure and death.^131,132^ Zuo et al.^133^ reported that a high viral load of HBV DNA is the critical correctable risk element in HCC recurrence. Researchers began to look at the correlations between sorafenib efficacy and viral reactivation, but opinions remain controversial. Two recently conducted studies reported that sorafenib blocked HCV infection by altering the viral entry step and the production of viral particles. In addition, sorafenib led to modification of claudin-1 expression and localization, which could partly be responsible for the anti-HCV effect.^134,135^ The opposite was shown in another study: combined application of sorafenib and interferon-α as an antiviral treatment was not beneficial.^136^ Moreover, Zhang and colleagues^137^ reported that sorafenib promoted virus reactivation by significantly reducing the number of natural killer (NK) cells and inhibiting the reactivity of NK cells against HCC cells. Lin et al.^120^ reported that targeting MYH9 noticeably promoted the survival of HCC-bearing mice and promoted sorafenib sensitivity of HCC cells in vivo. HBV X protein (HBX) interacted with MYH9 and triggered its expression through the modulation of GSK3β/β-catenin/c-Jun signaling. Witt-Kehati et al.^121^ reported that inhibition of pMAPK14 overturned resistance to sorafenib in hepatoma cells with HBV. The phosphorylated form of the pro-oncogenic protein mitogen-activated protein kinase 14 (pMAPK14) was triggered in sorafenib-treated hepatoma cells and related to HBV X protein expression. Therefore, there is no consensus on the correlation between viral reactivation and sorafenib therapy.

## Other possible mechanisms of sorafenib resistance in HCC

Some diverse studies have indicated that sorafenib suppresses the EMT process. KPNA3, KIAA1199, and CDK1 were markedly elevated in SR cells and positively related to a high risk of recurrence and metastasis and advanced TNM stage in patients with HCC. All of them were found to trigger EMT and are involved in EGFR phosphorylation and the AKT-ERK signaling cascade.^138–140^ Blocking these genes may improve sorafenib antitumor responses, providing a rational combination treatment to increase sorafenib efficacy (Table 5).^138,141–143^ It has been recently shown that cancer stem cells (CSCs) also participate in therapeutic resistance in HCC. CSC markers act as predictors in the response to sorafenib. Overexpression of the CSC markers CD90 and CD133 in HCC was related to a poorer response to sorafenib than normal expression of these markers (Table 5).^144^ Protein tyrosine kinase 2 (PTK2) was found to activate CSC traits and drive tumorigenicity by facilitating the nuclear accumulation of β-catenin in HCC cells, leading to sorafenib resistance.^144^ Xiao et al.^145^ reported that hypoxia is likely to enrich the HCC CSC population by changing AR/miR-520f-3p/SOX9 signaling, and targeting of this affected signaling by the small molecule miR-520f-3p could facilitate novel therapy to more effectively restrain HCC progression. These studies will help motivate researchers to explore sorafenib resistance and help develop effective strategies for the treatment of HCC in the clinic. Apart from this, several studies on sorafenib resistance in HCC have been reported (Table 5);^146–148^ however, the mechanism is not within the scope of this review. For example, Haga et al.^146^ found that overexpression of c-Jun contributed to sorafenib resistance in human hepatoma cell lines. The activation of JNK and high CD133 expression levels predicted an ineffective response to sorafenib in HCC.^147^ Downregulation of TGF-β expression reversed the sorafenib resistance of HCC cells.^148^

**Table 5:** Other factores and sorafenib resistance in HCC

Other factors				
KPNA3	Up	A novel KPNA3-AKT-ERK-TWIST signaling cascade promoted EMT and mediates sorafenib resistance in HCC	EMT	^138^
TNF-α	Up	Inhibiting the expression of TNF-α with ulinastatin significantly enhanced the anti-tumor effect of sorafenib on HCC cells with high expression of TNF-α in vitro and in vivo	EMT	^141^
Pin1	Up	Genetic or chemical Pin1 inhibition reversed Regorafenib resistance of HCC with reducing EMT, migration, invasion and metastasis	EMT	^142^
ADAM-17	up	ZLDI-8 treatment (a inhibitor for Notch activating/cleaving enzyme ADAM-17) enhanced the susceptibility of HCC cells to a small molecular kinase inhibitor sorafenib	EMT	^143^
PTK2	Up	PTK2 activates CSC traits and drives tumorigenicity in HCC cells, leading to HCC recurrence and sorafenib resistance	–	^144^
c-Jun		Regulation and phosphorylation of c-Jun can enhance cancer cell resistance to sorafenib	–	^146^
JNK		JNK activation positively correlated with increased expression of CD133, reducing cancer cell response to sorafenib treatment	–	^147^
TGF-β		By downregulating TGF-β, sorafenib inhibits phosphorylation of p38 and increases the sensitivity of HCC cells to sorafenib	–	^148^

## Strategies to overcome sorafenib resistance in HCC

### Combination of cytotoxic chemotherapeutic agents

Novel combination strategies including sorafenib and cytotoxic drugs have been studied to overcome resistance to sorafenib and to more effectively treat intermediate and advanced HCC. Wang et al.^149^ reported that sequential treatment with sorafenib and irinotecan was significantly better than monotherapy at inhibiting the growth of HepG2 xenografts. Sequential treatment with sorafenib and irinotecan noticeably elevated the levels of cleaved caspase-8, cleaved caspase-3, and PARP in HepG2 cells. Sorafenib inhibits p53 expression at both the mRNA and protein levels, which may facilitate cell cycle arrest and sensitization of tumor cells to irinotecan. Sequential therapy with sorafenib and irinotecan enhanced the efficacy of the two drugs alone in inducing apoptosis of HepG2 cells in vitro and inhibiting the growth of xenograft HepG2 cells in vivo. Although the addition of sorafenib to adriamycin did not significantly delay progression, the median survival time associated with sorafenib plus adriamycin was significantly longer than that associated with adriamycin alone in terms of overall survival and progression-free survival.^150^ In recent years, it has been reported that the liquid-gas phase transformation of sorafenib/doxorubicin-nanodroplets (SF/DOX-NDs) can be used as cavitation nuclei to promote drug release and increase cell uptake after therapeutic ultrasound (TUS) irradiation. In addition, this strategy can also induce the apoptosis of HCC cells and suppress HCC cell proliferation, migration and invasion, suggesting that SF/DOX-ND combination treatment may be a low-side effect and effective treatment for HCC.^151^ In addition, it was found that GEMOX based on sorafenib as a first-line medical treatment followed by sorafenib as maintenance therapy exhibited high performance with manageable toxicity for HCC patients in the late stage.^152^ Regarding the mechanism, a recent study showed that NOD2 made HCC cells noticeably more susceptible to sorafenib, lenvatinib and 5-FU treatment by activating the AMPK pathway to trigger apoptosis.^153^ Slit3 repression triggered chemoresistance to sorafenib, oxaliplatin and 5-FU via cyclin D3 and by enxtending survival and inhibiting β-catenin degradation.^154^ However, the different pharmacokinetic characteristics, hydrophobicity, and systemic toxicity of these drugs present serious challenges to the clinical application of this combination therapy. The efficacy and safety of some cytotoxic chemotherapy drugs are still being determined in phase III clinical trials, and the clinical use of cytotoxic chemotherapy drugs in combination with sorafenib requires further research.

### Combination of molecular targeted agents

#### Targeting EGFR

EGF and EGFR are critical to the development of HCC, and strategies targeting EGFR are able to overcome sorafenib resistance. In clinical practice, in addition to anti-EGFR antibodies (e.g., cetuximab), there are also TKIs targeting EGFR. According to some preclinical studies, cetuximab alone or in combination can inhibit HCC cell proliferation.^155–157^ Nevertheless, in one phase II study, cetuximab therapy did not show encouraging results.^158^ One study explored the performance of a combination of the anti-EGFR antibody cetuximab with oxaliplatin and capecitabine in late-stage HCC and showed that the capecitabine/oxaliplatin/cetuximab combination was tolerable, although there was a high rate of diarrhea. The combination was associated with only a modest response rate but a substantial α-fetoprotein (AFP) response and a high radiographically determined stable disease rate. The time to progression and overall survival were shorter than would be expected for treatment with sorafenib.^159^ In patients who received gemcitabine plus oxaliplatin (GEMOX) combined with cetuximab therapy, the response rate reached 20%, while 40% of patients developed stable disease.^160^ Erlotinib is an EGFR-specific receptor tyrosine kinase inhibitor. Disappointingly, a recent phase III randomized, double-blind, placebo-controlled trial showed that sorafenib in combination with erlotinib had no beneficial effect on survival in late-stage HCC patients compared to sorafenib alone.^161^

#### Targeting PI3K/AKT/mTOR signaling

Sorafenib suppresses the Raf/MAPK signaling pathway, whereas it can activate the PI3K/AKT pathway, suggesting an interaction between the MAPK/ERK pathway and the PI3K/AKT pathway. The potential compensation mechanism presented by the PI3K/AKT pathway can cause sorafenib resistance in HCC patients.^162,163^ Accordingly, by inhibiting the multiple pathways involved in HCC, a more effective survival outcome is likely to be achieved with a combination of targeted therapies. Liangtao Ye et al.^164^ found that copanlisib led to cell cycle arrest by affecting the cyclin D1/CDK4/6 signaling pathway, which significantly reduced cell activity and inhibited the colony forming process in various natural and sorafenib-resistant HCC cells. An increase in phosphorylated AKT was identified in cells being treated with sorafenib and was uniformly observed in 6 diverse clones of sorafenib-resistant cells that were not stimulated. Sorafenib plus copanlisib to treat HCC in the late stage is a reasonable potential therapy. Interestingly, the novel CDK4/6 inhibitors palbociclib and ribociclib, which were recently approved to treat hormone receptor-positive and HER2-negative breast cancer, both demonstrated antitumor activity in SR HCC cells and acted synergistically with sorafenib in HCC cell lines. Both agents induced cell cycle arrest in HCC cells that express Rb protein.^165,166^ Marozin et al.^167^ demonstrated that NSC74859, a specific inhibitor of signal transducer and activator of transcription 3 (STAT3), effectively inhibits the proliferation of HCC cells and is likely to be incorporated into vesicular stomatitis virus (VSV) oncolytic virus therapy. The protection of primary hepatocytes and nervous system cells from virus-triggered cytotoxic effects by NSC74859 increased the maximal tolerated dose of mouse VSV and enhanced the potential benefits of this combination therapy. The combination of sorafenib, which targets the PI3K/AKT/mTOR pathway, with PKI-587, which primarily targets the Ras/Raf/MAPK signaling pathway, outperformed single-agent therapy by blocking both signaling pathways.^168^ However, no evidence was found that sorafenib plus everolimus improves the efficacy compared with sorafenib alone. A combination of 5 mg everolimus with full-dose sorafenib is feasible but has been shown to be more toxic than sorafenib alone.^169^

### Combination with immunotherapeutic drugs

Immune checkpoint blockade is a promising treatment for HCC. Immunotherapy drugs cannot undergo liver metabolization, and HCC has moderate immunogenicity. For these reasons, from a theoretical perspective, the pharmacokinetic characteristics of immunotherapies make them rational choices in HCC that will not cause serious hepatotoxicity.^170^ Therefore, the combination of immunotherapy drugs and sorafenib to treat HCC in the late stage represents a novel therapeutic approach. Wang et al.^171^ reported that a combination of sorafenib and an anti-PD-L1 monoclonal antibody (mAb) can be used to treat HCC. According to Chen and colleagues,^114^ anti-PD-1 therapy enhanced the antitumor immune response of HCC models. Immunotherapy combinations containing anti-PD-1 antibodies plus sorafenib showed good performance only when they contained another drug to simultaneously target the immunosuppressive and hypoxic characteristics of the microenvironment (e.g., an additional CXCR4-inhibiting element). Recent in vivo and in vitro results suggest that targeting CD47 with combinations including sorafenib results in a beneficial effect on patients with HCC.^172^ However, the effective use immunotherapy in HCC must fully consider the HCC-specific immune microenvironment and response.

### Conclusions and future perspectives

Resistance to systemic sorafenib therapy emerges early in HCC. To enhance the antitumor effect induced by sorafenib, it is urgent to understand its potential mechanism and identify therapeutic targets. This study comprehensively summarized the molecular, cellular, and microenvironmental mechanisms that are likely to collectively facilitate sorafenib resistance in HCC. Epigenetic biological processes, transport processes, regulated cell death, and the tumor microenvironment have been shown to be related to resistance to sorafenib. To maintain the efficacy of sorafenib against HCC, we need to examine treatments for initial or acquired drug resistance.
